# Supplementary material for: A Likelihood Approach for Real-Time Calibration of Stochastic Compartmental Epidemic Models
Source: PLoS Comput Biol. 2017 Jan 17;13(1):e1005257. doi: 10.1371/journal.pcbi.1005257 (PMC5240920; doi:10.1371/journal.pcbi.1005257)
Supplement: S1 File — (TAR.GZ) [file pcbi.1005257.s014.tar.gz › HSPH_Online-SI-Revision/MSS10/n100-severe/n100-severe_NEW2_table4.pdf]

1	newly 3 weeks spec	1675.	1971.86	{1902., 2040.}	17.723	0.0706923	newly 3 weeks cumul	7166.	8098.	{7968., 8215.}	13.0059	0.0530655	newly 5 weeks spec	875.	1043.47	{985., 1106.}	19.2537	0.0760629	newly 5 weeks cumul	9240.	10 577.3	{10 398., 10 756.}	14.4725	0.0586614	newly 46 weeks cumul	11 305.	13 207.	{12 949., 13 439.}	16.8241	0.0674761
2	newly 3 weeks spec	260.	331.82	{264., 391.}	28.6692	0.105776	newly 3 weeks cumul	1782.	2010.16	{1791., 2197.}	13.2278	0.052863	newly 5 weeks spec	65.	101.43	{66., 133.}	57.1846	0.184367	newly 5 weeks cumul	1949.	2298.15	{2045., 2533.}	18.0241	0.070503	newly 53 weeks cumul	2047.	2415.59	{2137., 2671.}	18.2096	0.0710975
3	newly 3 weeks spec	220.	154.43	{123., 188.}	29.8045	0.159362	newly 3 weeks cumul	1526.	1208.57	{1114., 1320.}	20.8014	0.10222	newly 5 weeks spec	41.	32.81	{16., 48.}	26.8049	0.150988	newly 5 weeks cumul	1670.	1314.09	{1194., 1424.}	21.312	0.105141	newly 54 weeks cumul	1741.	1343.13	{1214., 1468.}	22.853	0.113799
4	newly 3 weeks spec	1102.	1324.91	{1245., 1394.}	20.2278	0.0796097	newly 3 weeks cumul	5564.	6298.56	{6127., 6445.}	13.202	0.0537676	newly 5 weeks spec	463.	567.56	{515., 619.}	22.5832	0.0872776	newly 5 weeks cumul	6701.	7737.01	{7502., 7947.}	15.4605	0.0623261	newly 52 weeks cumul	7579.	8758.94	{8467., 9072.}	15.5685	0.0626826
5	newly 3 weeks spec	2014.	218.24	{156., 290.}	89.1639	0.977368	newly 3 weeks cumul	7895.	2678.77	{2336., 3022.}	66.07	0.471539	newly 5 weeks spec	1193.	26.54	{6., 50.}	97.7754	1.76775	newly 5 weeks cumul	10 658.	2779.93	{2380., 3143.}	73.917	0.585979	newly 46 weeks cumul	14 294.	2794.48	{2401., 3146.}	80.45	0.711272
6	newly 3 weeks spec	1306.	1389.99	{1317., 1453.}	6.67764	0.0278202	newly 3 weeks cumul	6189.	6558.6	{6392., 6741.}	5.97189	0.0251008	newly 5 weeks spec	562.	601.13	{555., 649.}	7.89858	0.0327025	newly 5 weeks cumul	7603.	8079.31	{7874., 8294.}	6.26476	0.0262927	newly 52 weeks cumul	8616.	9188.22	{8941., 9430.}	6.64136	0.0278222
7	newly 3 weeks spec	678.	810.04	{752., 866.}	19.4749	0.0766494	newly 3 weeks cumul	3914.	4712.78	{4544., 4869.}	20.4083	0.0804825	newly 5 weeks spec	239.	258.27	{218., 292.}	11.5858	0.0471208	newly 5 weeks cumul	4550.	5430.87	{5220., 5605.}	19.3598	0.0766607	newly 53 weeks cumul	4820.	5756.08	{5515., 5991.}	19.4207	0.0768373
8	newly 3 weeks spec	1197.	857.43	{777., 937.}	28.3684	0.146083	newly 3 weeks cumul	5691.	4272.17	{4028., 4488.}	24.9311	0.124905	newly 5 weeks spec	560.	338.21	{285., 386.}	39.6054	0.221861	newly 5 weeks cumul	7047.	5149.	{4840., 5431.}	26.9334	0.136714	newly 49 weeks cumul	8398.	5715.03	{5394., 6043.}	31.9477	0.167634
9	newly 3 weeks spec	121.	147.87	{110., 185.}	26.7355	0.0997575	newly 3 weeks cumul	1328.	1371.04	{1181., 1527.}	8.86446	0.0374681	newly 5 weeks spec	4.	25.75	{10., 42.}	544.25	0.745086	newly 5 weeks cumul	1373.	1457.3	{1255., 1634.}	10.2797	0.0424211	newly 54 weeks cumul	1373.	1475.24	{1268., 1672.}	11.0153	0.0450046
10	newly 3 weeks spec	840.	669.43	{592., 720.}	20.306	0.0997342	newly 3 weeks cumul	4139.	3739.59	{3497., 3946.}	9.64992	0.0445368	newly 5 weeks spec	240.	227.64	{189., 268.}	11.15	0.0513028	newly 5 weeks cumul	4837.	4359.27	{4104., 4585.}	9.87658	0.0456311	newly 47 weeks cumul	5228.	4662.12	{4350., 4916.}	10.824	0.0502502
11	newly 3 weeks spec	467.	564.23	{511., 615.}	20.8458	0.0809416	newly 3 weeks cumul	2925.	3301.94	{3110., 3461.}	12.8868	0.0522446	newly 5 weeks spec	130.	178.2	{143., 215.}	37.3846	0.132864	newly 5 weeks cumul	3288.	3794.92	{3576., 4003.}	15.4173	0.0618499	newly 53 weeks cumul	3458.	4021.88	{3781., 4254.}	16.3065	0.0651111
12	newly 3 weeks spec	1748.	2051.25	{1414., 2414.}	28.8896	0.113804	newly 3 weeks cumul	7573.	8138.91	{6234., 9147.}	18.729	0.0787718	newly 5 weeks spec	887.	1178.59	{694., 1463.}	45.566	0.164809	newly 5 weeks cumul	9747.	10 875.7	{7931., 12 407.}	23.3002	0.0953508	newly 46 weeks cumul	12 152.	14 328.7	{9458., 16 912.}	31.8083	0.125725
13	newly 3 weeks spec	1801.	1757.29	{1689., 1829.}	3.24986	0.0144174	newly 3 weeks cumul	7188.	7314.89	{7166., 7491.}	2.00403	0.00858782	newly 5 weeks spec	954.	918.39	{868., 970.}	4.66352	0.0209141	newly 5 weeks cumul	9478.	9515.26	{9309., 9727.}	1.30935	0.00566224	newly 46 weeks cumul	11 854.	11 830.2	{11 521., 12 113.}	1.52649	0.00663988
14	newly 3 weeks spec	2332.	2448.29	{2348., 2543.}	5.26715	0.0221396	newly 3 weeks cumul	8946.	9732.97	{9526., 9928.}	8.79689	0.0365604	newly 5 weeks spec	1422.	1357.54	{1291., 1433.}	4.98172	0.0224147	newly 5 weeks cumul	12 296.	12 921.7	{12 663., 13 214.}	5.10719	0.021576	newly 46 weeks cumul	16 703.	16 424.5	{16 048., 16 847.}	2.0549	0.00904839
15	newly 3 weeks spec	957.	919.48	{834., 1002.}	6.45977	0.0291992	newly 3 weeks cumul	4781.	4484.58	{4158., 4762.}	6.65802	0.0302886	newly 5 weeks spec	410.	372.8	{306., 435.}	11.7854	0.0556356	newly 5 weeks cumul	5823.	5441.41	{5064., 5809.}	6.97802	0.0317908	newly 48 weeks cumul	6577.	6069.85	{5608., 6440.}	8.01444	0.0367232
16	newly 3 weeks spec	2249.	2163.	{2074., 2235.}	4.0418	0.0180749	newly 3 weeks cumul	9354.	8500.92	{8316., 8687.}	9.11995	0.0415877	newly 5 weeks spec	1205.	1224.13	{1155., 1301.}	3.59585	0.0153734	newly 5 weeks cumul	12 257.	11 364.7	{11 098., 11 593.}	7.28017	0.0328862	newly 47 weeks cumul	15 533.	14 693.8	{14 345., 15 003.}	5.4025	0.0241953
17	newly 3 weeks spec	539.	420.88	{342., 500.}	22.5603	0.11532	newly 3 weeks cumul	2954.	2728.83	{2389., 3077.}	9.6063	0.0446776	newly 5 weeks spec	198.	112.4	{73., 159.}	43.2323	0.266956	newly 5 weeks cumul	3479.	3065.96	{2689., 3510.}	12.7864	0.0609462	newly 53 weeks cumul	3686.	3191.89	{2721., 3656.}	14.1555	0.0680715
18	newly 3 weeks spec	789.	919.74	{834., 994.}	16.6793	0.0660755	newly 3 weeks cumul	4078.	4504.39	{4302., 4702.}	10.474	0.0429891	newly 5 weeks spec	352.	373.97	{323., 426.}	10.4574	0.0430867	newly 5 weeks cumul	4968.	5469.98	{5190., 5742.}	10.1876	0.0418302	newly 51 weeks cumul	5544.	6107.79	{5746., 6416.}	10.3032	0.042262
19	newly 3 weeks spec	457.	441.54	{387., 491.}	7.77243	0.0351052	newly 3 weeks cumul	2528.	2526.9	{2363., 2702.}	4.15981	0.0181188	newly 5 weeks spec	174.	144.62	{114., 173.}	18.6552	0.093782	newly 5 weeks cumul	2969.	2926.84	{2730., 3149.}	4.53688	0.0200298	newly 51 weeks cumul	3262.	3122.08	{2894., 3343.}	5.65543	0.0255803
20	newly 3 weeks spec	674.	616.83	{554., 692.}	9.6632	0.0448139	newly 3 weeks cumul	3591.	3375.49	{3185., 3549.}	6.20635	0.028169	newly 5 weeks spec	266.	215.86	{180., 251.}	19.2632	0.0959438	newly 5 weeks cumul	4252.	3954.39	{3750., 4160.}	7.13711	0.0325347	newly 49 weeks cumul	4608.	4259.4	{4016., 4508.}	7.73394	0.0353441
21	newly 3 weeks spec	521.	608.88	{556., 665.}	16.9021	0.0667008	newly 3 weeks cumul	3210.	3579.34	{3414., 3738.}	11.5059	0.047023	newly 5 weeks spec	157.	188.85	{152., 225.}	22.465	0.0856555	newly 5 weeks cumul	3675.	4112.22	{3910., 4324.}	11.8971	0.0484864	newly 53 weeks cumul	3774.	4344.36	{4103., 4596.}	15.1129	0.0607052
22	newly 3 weeks spec	558.	536.82	{457., 600.}	8.40143	0.0381441	newly 3 weeks cumul	3543.	3264.52	{2984., 3551.}	8.22523	0.0380178	newly 5 weeks spec	178.	155.44	{115., 196.}	18.2809	0.0907821	newly 5 weeks cumul	4026.	3711.08	{3408., 4031.}	8.24441	0.0381291	newly 51 weeks cumul	4274.	3892.21	{3579., 4228.}	9.27679	0.0431335
23	newly 3 weeks spec	35.	51.26	{31., 74.}	52.1143	0.171552	newly 3 weeks cumul	468.	512.08	{398., 617.}	16.5385	0.066781	newly 5 weeks spec	5.	7.09	{1., 16.}	88.6	0.391198	newly 5 weeks cumul	490.	538.7	{415., 644.}	17.0163	0.068538	newly 55 weeks cumul	497.	544.05	{415., 655.}	16.9034	0.0682982
24	newly 3 weeks spec	330.	262.92	{218., 306.}	20.5636	0.103433	newly 3 weeks cumul	2438.	1983.54	{1797., 2135.}	18.6407	0.0904965	newly 5 weeks spec	72.	58.9	{40., 78.}	22.6667	0.120171	newly 5 weeks cumul	2664.	2168.57	{1947., 2339.}	18.5972	0.0903351	newly 56 weeks cumul	2695.	2223.13	{2002., 2408.}	17.5091	0.084604
25	newly 3 weeks spec	192.	126.44	{79., 170.}	35.125	0.202285	newly 3 weeks cumul	1247.	1125.65	{910., 1324.}	12.8685	0.0618855	newly 5 weeks spec	31.	21.86	{7., 39.}	44.0645	0.307063	newly 5 weeks cumul	1365.	1200.26	{963., 1389.}	14.1993	0.0694158	newly 56 weeks cumul	1367.	1217.1	{968., 1428.}	13.8727	0.0674476
26	newly 3 weeks spec	830.	716.26	{650., 777.}	13.7036	0.0649581	newly 3 weeks cumul	4614.	3943.22	{3797., 4099.}	14.5379	0.0684273	newly 5 weeks spec	289.	249.72	{214., 283.}	14.	0.0675071	newly 5 weeks cumul	5390.	4614.69	{4435., 4803.}	14.3842	0.067641	newly 50 weeks cumul	5805.	4964.15	{4759., 5161.}	14.4849	0.0681655
27	newly 3 weeks spec	578.	518.61	{459., 576.}	10.8218	0.050942	newly 3 weeks cumul	3242.	3069.06	{2901., 3230.}	5.83282	0.0263559	newly 5 weeks spec	170.	155.78	{116., 190.}	14.8118	0.0711315	newly 5 weeks cumul	3757.	3513.58	{3288., 3729.}	6.84908	0.031179	newly 51 weeks cumul	4031.	3716.08	{3489., 3953.}	7.96874	0.0365082
28	newly 3 weeks spec	1075.	942.46	{836., 1029.}	12.4316	0.0588772	newly 3 weeks cumul	4677.	4335.8	{4091., 4555.}	7.37823	0.0336887	newly 5 weeks spec	430.	425.06	{368., 483.}	8.44651	0.0370765	newly 5 weeks cumul	5845.	5395.14	{5046., 5717.}	7.83131	0.0358856	newly 50 weeks cumul	7034.	6232.95	{5833., 6653.}	11.4602	0.053421
29	newly 3 weeks spec	909.	1118.87	{1067., 1177.}	23.088	0.0899304	newly 3 weeks cumul	4464.	5348.12	{5206., 5484.}	19.8056	0.0783936	newly 5 weeks spec	364.	476.35	{431., 521.}	30.8654	0.115618	newly 5 weeks cumul	5336.	6555.05	{6345., 6723.}	22.8458	0.0892618	newly 51 weeks cumul	5817.	7425.48	{7186., 7642.}	27.6514	0.105894
30	newly 3 weeks spec	1036.	1048.39	{962., 1144.}	5.29633	0.022706	newly 3 weeks cumul	4857.	4866.9	{4619., 5129.}	2.99691	0.0129869	newly 5 weeks spec	473.	463.82	{392., 536.}	9.09091	0.0404463	newly 5 weeks cumul	6087.	6030.37	{5686., 6364.}	3.39724	0.0148912	newly 47 weeks cumul	7069.	6916.19	{6484., 7325.}	4.14047	0.0183614
31	newly 3 weeks spec	35.	36.15	{16., 57.}	37.6857	0.172719	newly 3 weeks cumul	499.	427.6	{308., 548.}	19.519	0.0989119	newly 5 weeks spec	17.	4.8	{0., 13.}	75.4118	1.11959	newly 5 weeks cumul	538.	446.27	{322., 576.}	21.5074	0.110763	newly 56 weeks cumul	540.	447.88	{322., 577.}	21.4926	0.110674
32	newly 3 weeks spec	307.	298.57	{248., 349.}	10.5179	0.0471417	newly 3 weeks cumul	2113.	2132.84	{1975., 2294.}	5.292	0.0228122	newly 5 weeks spec	67.	70.34	{48., 94.}	22.3881	0.0956086	newly 5 weeks cumul	2352.	2349.22	{2153., 2563.}	5.56207	0.0242166	newly 54 weeks cumul	2475.	2411.59	{2179., 2625.}	6.11192	0.0272219
33	newly 3 weeks spec	386.	328.42	{282., 383.}	15.5389	0.0755999	newly 3 weeks cumul	2219.	2038.12	{1922., 2163.}	8.28391	0.0380239	newly 5 weeks spec	106.	92.49	{71., 115.}	17.5943	0.0861398	newly 5 weeks cumul	2533.	2307.29	{2151., 2454.}	9.03158	0.0416848	newly 54 weeks cumul	2695.	2409.62	{2235., 2577.}	10.6649	0.049629
34	newly 3 weeks spec	1133.	665.92	{596., 738.}	41.2251	0.232414	newly 3 weeks cumul	5334.	3635.44	{3307., 3888.}	31.844	0.1674	newly 5 weeks spec	504.	231.14	{191., 271.}	54.1389	0.34291	newly 5 weeks cumul	6608.	4260.42	{3907., 4591.}	35.5263	0.191529	newly 46 weeks cumul	7621.	4590.35	{4241., 4930.}	39.7671	0.221061
35	newly 3 weeks spec	193.	123.69	{91., 158.}	35.9119	0.202253	newly 3 weeks cumul	1207.	1138.15	{1007., 1283.}	9.35294	0.0433359	newly 5 weeks spec	21.	21.87	{8., 37.}	46.619	0.217567	newly 5 weeks cumul	1310.	1212.7	{1054., 1389.}	10.1756	0.047929	newly 55 weeks cumul	1313.	1228.51	{1060., 1404.}	9.97334	0.0466206
36	newly 3 weeks spec	618.	448.09	{399., 497.}	27.4935	0.14124	newly 3 weeks cumul	3733.	3099.71	{2928., 3278.}	16.9646	0.0811546	newly 5 weeks spec	195.	112.95	{87., 144.}	42.1385	0.246671	newly 5 weeks cumul	4250.	3438.08	{3236., 3652.}	19.104	0.0925591	newly 53 weeks cumul	4446.	3553.25	{3338., 3806.}	20.0798	0.0978998
37	newly 3 weeks spec	114.	116.16	{76., 163.}	23.1053	0.101369	newly 3 weeks cumul	920.	942.47	{728., 1120.}	13.4793	0.0582466	newly 5 weeks spec	26.	22.52	{8., 37.}	37.7692	0.214882	newly 5 weeks cumul	1039.	1017.59	{802., 1225.}	13.3041	0.0600782	newly 55 weeks cumul	1048.	1037.95	{813., 1246.}	13.501	0.0601601
38	newly 3 weeks spec	477.	511.93	{463., 558.}	8.60587	0.0352456	newly 3 weeks cumul	2807.	3170.25	{3030., 3329.}	12.9409	0.0525976	newly 5 weeks spec	136.	148.58	{117., 173.}	14.6471	0.0591788	newly 5 weeks cumul	3197.	3596.17	{3437., 3777.}	12.4858	0.0507936	newly 53 weeks cumul	3359.	3770.26	{3591., 3990.}	12.2435	0.0498184
39	newly 3 weeks spec	862.	721.2	{609., 809.}	16.4524	0.080196	newly 3 weeks cumul	4078.	3542.61	{3191., 3874.}	13.1709	0.0624645	newly 5 weeks spec	352.	291.48	{231., 351.}	18.6989	0.0939332	newly 5 weeks cumul	4972.	4292.75	{3777., 4720.}	13.7263	0.0654001	newly 50 weeks cumul	5595.	4797.6	{4184., 5300.}	14.3478	0.0688877
40	newly 3 weeks spec	607.	587.7	{519., 656.}	7.95717	0.0355502	newly 3 weeks cumul	3292.	3187.52	{2932., 3400.}	5.28433	0.0236958	newly 5 weeks spec	210.	206.64	{165., 246.}	11.819	0.0525536	newly 5 weeks cumul	3890.	3742.59	{3430., 4001.}	5.46093	0.0246196	newly 53 weeks cumul	4289.	4040.74	{3681., 4317.}	6.64677	0.0303641
41	newly 3 weeks spec	231.	253.47	{212., 294.}	13.5022	0.0547473	newly 3 weeks cumul	1656.	1738.95	{1591., 1899.}	6.7808	0.028241	newly 5 weeks spec	65.	62.87	{42., 82.}	17.4615	0.0798778	newly 5 weeks cumul	1848.	1929.42	{1750., 2125.}	6.61472	0.0276536	newly 55 weeks cumul	1919.	1991.15	{1805., 2182.}	6.4987	0.0272999
42	newly 3 weeks spec	1150.	815.72	{730., 887.}	29.0678	0.150316	newly 3 weeks cumul	5514.	4229.94	{3994., 4458.}	23.2873	0.1155	newly 5 weeks spec	480.	296.37	{250., 345.}	38.2563	0.212748	newly 5 weeks cumul	6798.	5019.41	{4761., 5282.}	26.1634	0.132164	newly 48 weeks cumul	7662.	5481.39	{5138., 5818.}	28.4601	0.145964
43	newly 3 weeks spec	1025.	856.41	{772., 963.}	16.5922	0.0802017	newly 3 weeks cumul	4521.	3979.26	{3707., 4239.}	12.0226	0.0561758	newly 5 weeks spec	563.	374.05	{312., 452.}	33.5613	0.18188	newly 5 weeks cumul	5852.	4921.51	{4560., 5217.}	15.9004	0.0758916	newly 47 weeks cumul	6825.	5657.75	{5227., 6122.}	17.1026	0.0822619
44	newly 3 weeks spec	487.	370.32	{317., 422.}	23.9589	0.121638	newly 3 weeks cumul	3260.	2635.37	{2419., 2812.}	19.1604	0.0930841	newly 5 weeks spec	138.	83.96	{60., 110.}	39.3768	0.228998	newly 5 weeks cumul	3654.	2895.61	{2650., 3079.}	20.7551	0.101803	newly 54 weeks cumul	3820.	2971.11	{2707., 3187.}	22.2223	0.109955
45	newly 3 weeks spec	960.	757.71	{669., 837.}	21.0719	0.104186	newly 3 weeks cumul	5111.	4257.84	{4009., 4516.}	16.6926	0.0797084	newly 5 weeks spec	424.	250.49	{201., 294.}	40.9222	0.232846	newly 5 weeks cumul	6116.	4946.49	{4643., 5264.}	19.1221	0.0926611	newly 52 weeks cumul	6740.	5276.34	{4912., 5634.}	21.716	0.106878
46	newly 3 weeks spec	833.	800.68	{733., 861.}	5.5078	0.0248159	newly 3 weeks cumul	4606.	4578.39	{4340., 4787.}	2.8465	0.0124466	newly 5 weeks spec	254.	257.07	{219., 300.}	9.39764	0.0405093	newly 5 weeks cumul	5399.	5289.39	{5009., 5527.}	3.2845	0.0145507	newly 51 weeks cumul	5711.	5614.43	{5313., 5891.}	3.40098	0.0150144
47	newly 3 weeks spec	1599.	2088.12	{2019., 2159.}	30.5891	0.115738	newly 3 weeks cumul	6760.	8031.12	{7901., 8161.}	18.8036	0.0747878	newly 5 weeks spec	834.	1248.66	{1181., 1306.}	49.7194	0.174972	newly 5 weeks cumul	8781.	10 899.9	{10 716., 11 056.}	24.131	0.093839	newly 47 weeks cumul	10 888.	14 753.5	{14 414., 15 085.}	35.5026	0.131881
48	newly 3 weeks spec	1207.	1452.75	{1385., 1529.}	20.3604	0.0801457	newly 3 weeks cumul	5943.	6873.08	{6690., 7035.}	15.65	0.0630428	newly 5 weeks spec	486.	618.18	{576., 659.}	27.1975	0.103764	newly 5 weeks cumul	7196.	8443.92	{8177., 8646.}	17.3419	0.0693492	newly 52 weeks cumul	7925.	9565.22	{9301., 9810.}	20.6968	0.0815835
49	newly 3 weeks spec	149.	101.15	{73., 133.}	32.651	0.182435	newly 3 weeks cumul	1122.	967.88	{832., 1134.}	14.8021	0.0720715	newly 5 weeks spec	19.	15.16	{4., 27.}	44.5263	0.27847	newly 5 weeks cumul	1199.	1022.46	{860., 1202.}	15.7314	0.0770703	newly 56 weeks cumul	1212.	1031.9	{860., 1212.}	15.9587	0.0782523
50	newly 3 weeks spec	163.	149.56	{115., 182.}	14.5399	0.0692078	newly 3 weeks cumul	1147.	1155.62	{996., 1286.}	8.13078	0.0352882	newly 5 weeks spec	19.	29.62	{15., 47.}	65.7895	0.211815	newly 5 weeks cumul	1229.	1252.28	{1077., 1397.}	8.67535	0.0372177	newly 55 weeks cumul	1262.	1276.72	{1090., 1430.}	8.52298	0.0367701
